# Insights into the Role of Vitamin C in Stabilizing Organic and Perovskite Solar Cells

**DOI:** 10.1021/acsami.4c22532

**Published:** 2025-02-18

**Authors:** Guan-Lin Chen, Kai-Wei Tseng, Ching-I Huang, Leeyih Wang

**Affiliations:** a Institute of Polymer Science and Engineering, National Taiwan University, Taipei 10617, Taiwan; b Center for Condensed Matter Sciences, National Taiwan University, Taipei 10617, Taiwan; c Center of Atomic Initiative for New Materials, National Taiwan University, Taipei 10617, Taiwan

**Keywords:** vitamin C, eco-friendly, interfacial interaction, robust interface, stability

## Abstract

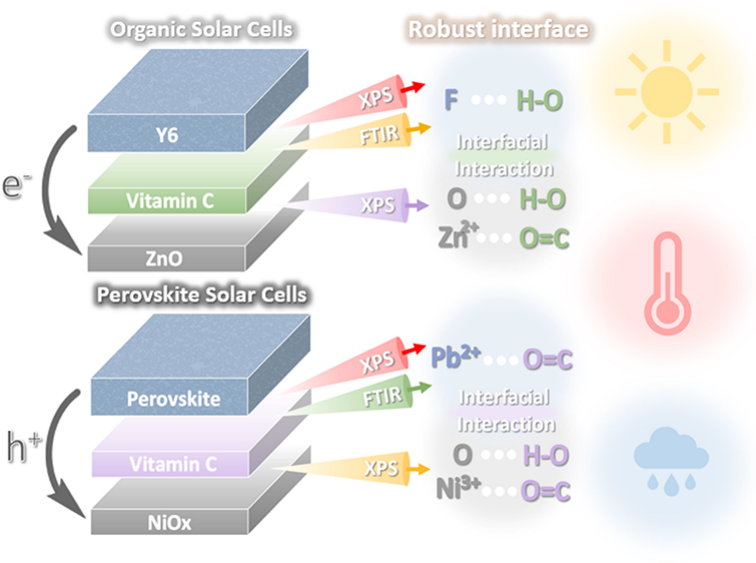

This study provides an in-depth exploration of the mechanisms by which vitamin C enhances interfacial stability in organic solar cells (OSCs) and perovskite solar cells (PSCs). In OSCs, vitamin C interacts with ZnO (O–H···O and C=O···Zn^2+^) and Y6 (O–H···F), forming a robust interface. The ZnO/vitamin C devices maintained 80% of their original efficiency (T_80_ lifetime) for 4437 h for PM6:Y6 (binary) and 6028 h for PM6:Y6:PC_71_BM (ternary) at 65 °C in a N_2_ atmosphere. Under AM1.5G one-sun illumination in a N_2_ atmosphere, the binary devices maintained 85% of their efficiency for 2100 h, while the ternary devices had a *T*_80_ lifetime of 1,680 h. In PSCs, vitamin C stabilized the NiO_*x*_ (O–H···O, C=O···Ni^3+^) and perovskite layer (C=O···Pb^2+^), achieving a *T*_80_ lifetime of 1198 h at 65 °C in a N_2_ atmosphere. These results demonstrate that vitamin C, as an interfacial stabilizer, offers a universal strategy to improve the practicality of photovoltaic devices.

## Introduction

Renewable energy technologies have been a subject of intense research, and among them, organic solar cells (OSCs) and perovskite solar cells (PSCs) have attracted considerable interest. These solar cells offer potential advantages such as flexibility, lightweight construction, solution processability, and low manufacturing costs for large-scale production.^1−6^ Moreover, substantial advancements in power conversion efficiency (PCE) have pushed OSCs and PSCs to impressive levels of 20%^7^ and 26%,^8^ respectively.

Inorganic oxide materials, such as zinc oxide (ZnO) and tin oxide (SnO_2_), are commonly used as electron transport layers (ETLs) in inverted OSC devices owing to their advantages in efficient electron extraction, eco-friendliness, high transparency, low-temperature processability, and cost-effectiveness.^9^ However, the inorganic oxide ETL can act as photocatalysts, reacting with adsorbed water and oxygen to generate free radicals (e.g., •OH and •O^2–^) upon irradiation with UV light.^10,11^ For nonfullerene acceptors (NFAs), the vinyl group of the enone in ITIC- and Y6-based derivatives is susceptible to radical-induced cleavage, breaking the double bond between the electron-donating and electron-accepting units.^12,13^ This disruption of molecular structure hinders electron transport at the inorganic oxide ETL/active layer interface. To address this issue, Balasubramanian et al. introduced vitamin C as a photostabilizer in inverted PBDB-T:IT-4F OSCs, where it functioned as an antioxidant interlayer at the ZnO ETL/active layer interface. This effectively suppressed ZnO’s photocatalytic activity and mitigated NFA photodegradation. After 96 h of standard one-sun AM1.5G illumination, reference devices lost 64% of their efficiency, whereas vitamin C devices lost only 38%.^14^

Nickel oxide (NiO_*x*_) is a widely used, low-cost hole transport material (HTM) in PSCs, offering good conductivity, high transparency, and low-temperature solution processability.^15,16^ However, the NiO_*x*_/perovskite interface can induce instability in the perovskite layer due to interfacial chemical reactions.^17−19^ The presence of Ni^3+^ ions in NiO_*x*_ imparts strong oxidizing and basic properties, which can lead to the deprotonation of methylammonium (MA^+^) and subsequent redox reactions between Ni^3+^ ions in NiO_*x*_ and I^–^ ions in the perovskite during thermal annealing.^17−19^ These reactions can cause perovskite lattice deformation, interface defects, and hole extraction barriers, further accelerating degradation upon exposure to H_2_O/O_2_.^17−20^

Recently, biomaterials have gained significant attention in solar cell applications due to their safety, biodegradability, nontoxicity, eco-friendliness, and sustainability.^21−23^ Their natural abundance further enhances cost-effectiveness and helps lower production expenses. Ascorbic acid, also referred to as vitamin C, is an organic compound naturally present in plants and fruits, exhibiting antioxidant properties that make it a crucial antioxidant in the human body.^24^ Vitamin C is a potent antioxidant and an efficient neutralizer of free radicals, effectively eliminating harmful oxygen-derived species in biological systems.^24,25^

This study explores the mechanism by which the vitamin C acts as a universal interfacial stabilizer. In OSCs, we demonstrate that vitamin C forms interactions with ZnO (O–H···O and C = O···Zn^2+^) and Y6 (O–H···F), thereby enhancing interfacial stability. The PM6:Y6 (binary) and PM6:Y6:PC_71_BM (ternary) systems with ZnO/vitamin C ETL retained 80% of their original performance (T_80_ lifetime) after 4,437 and 6,028 h at 65 °C in an N_2_ atmosphere, respectively. Moreover, these binary and ternary devices utilizing ZnO/vitamin C ETL exhibit exceptional long-term stability under continuous one-sun AM1.5G illumination in an N_2_ environment. The binary device preserves 85% of its initial PCE after 2,100 h, while the ternary device achieves a T_80_ lifetime of 1,680 h. In PSCs, vitamin C strengthens the stability of the interface between NiO_*x*_ (O–H···O and C = O···Ni^3+^) and the perovskite layer (C = O···Pb^2+^). Our study reveals that using NiO_*x*_/vitamin C as the HTM resulted in exceptional durability, with a T_80_ lifetime of 1,198 h, even after thermal aging at 65 °C in an N_2_ environment.

## Results and Discussion

**Figure 1:**
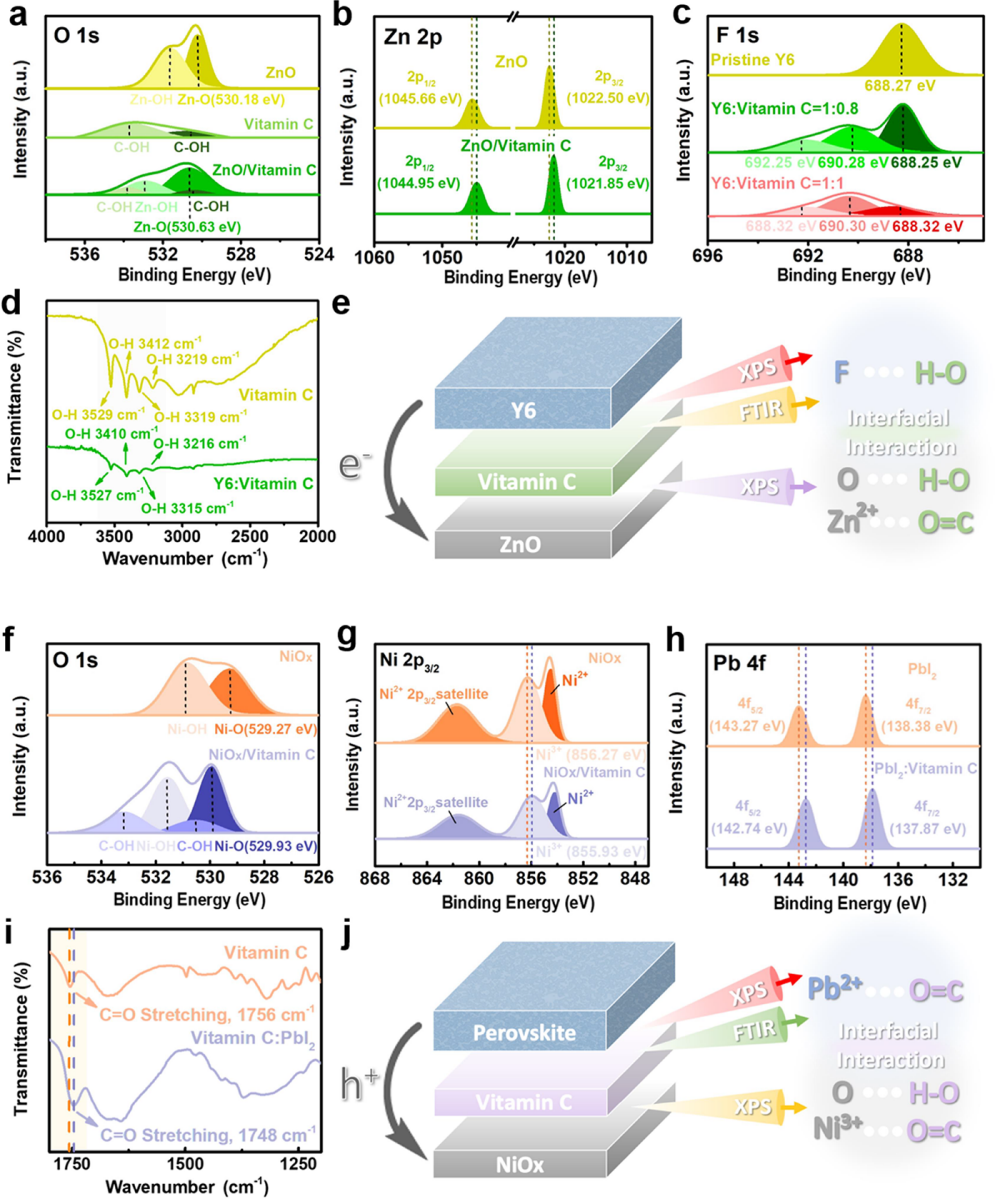
XPS spectra: (a) O 1s and (b) Zn 2p for ZnO and ZnO/vitamin C films; (c) F 1s for Y6 and the Y6:vitamin C mixture at different molar ratios; (d) FTIR spectra for vitamin C and the Y6:vitamin C mixture; (e) schematic illustration of the interaction between vitamin C, Y6, and ZnO. XPS spectra: (f) O 1s and (g) Ni 2p for NiO_*x*_ and NiO_*x*_/vitamin C HTMs; (h) Pb 4f for PbI_2_ and the PbI_2_:vitamin C mixture (1:1 molar ratio). (i) FTIR spectra for vitamin C and the vitamin C:PbI_2_ mixture (1:1 molar ratio). (j) Schematic illustration of the interaction between vitamin C, NiO_*x*_, and perovskite.

X-ray photoelectron spectroscopy (XPS) and Fourier-transform infrared (FTIR) analyses first investigate vitamin C’s interaction with the charge transport and photoactive layers. In Figure 1a, the XPS spectrum of the ZnO film displays two distinct O 1s peaks located at 531.62 and 530.18 eV, aligning with oxygen in the Zn–OH group and Zn–O, respectively. The O 1s peaks for vitamin C are observed at 533.70 and 530.48 eV, respectively. The Zn–O O 1s peak shifts from 530.20 to 530.64 eV in the ZnO/vitamin C film, indicating hydrogen bonding (O–H···O) between vitamin C and ZnO. Figure 1b presents the XPS spectrum of the ZnO film, which exhibits two distinct peaks at 1022.50 and 1045.66 eV in the Zn 2p region, corresponding to the 2p_3/2_ and 2p_1/2_ orbitals, respectively. Changes in the Zn 2p peaks are also observed for the ZnO/vitamin C film, shifting to 1021.85 and 1044.95 eV. These data suggest that vitamin C can not only interact with ZnO through O–H···O bonds but also coordinate with zinc atoms via C = O···Zn^2+^ interactions. These results suggest a significant interaction between vitamin C and ZnO. In Figure 1c, the XPS spectrum of Y6 shows an F 1s peak located at 688.27 eV. As the molar ratio of vitamin C in the Y6:vitamin C mixture increases, the F 1s peak position of Y6 shifts to higher binding energies. Specifically, in the 1:0.8 mixture, the peak positions are 688.25, 690.28, and 692.25 eV, while in the 1:1 mixture, the peak positions are 688.32, 690.30, and 692.32 eV. The original peak intensity ratio of Y6 gradually decreases with the increasing vitamin C ratio. These results suggest the existence of O–H···F interactions at the vitamin C/Y6 interface, further supported by the FTIR spectrum in Figure 1d. The O–H stretching peaks of vitamin C appear at 3219, 3319, 3412, and 3529 cm^–1^. In the Y6:vitamin C (1:1) mixture, these peaks shift to 3216, 3315, 3410, and 3527 cm^–1^, due to the high electronegativity of F atoms in Y6, which elongates the O–H bond and lowers the stretching frequency. These results indicate an interaction between vitamin C and both ZnO (O–H···O and C = O···Zn^2+^) and Y6 (O–H···F), as shown in Figure 1e.

Furthermore, we continued to investigate the interactions between vitamin C, NiO_*x*_, and Perovskite. As shown in Figure 1f, the O 1s peaks in the NiO_*x*_ film XPS spectrum at 529.27 and 530.92 eV are attributed to oxygen in the Ni–OH group and Ni–O, respectively. In the NiO_*x*_/vitamin C film, the O 1s Ni–O peak shifts to a higher binding energy of 529.93 eV, indicating the presence of strong hydrogen bonding (O–H···O) between vitamin C and the oxygen atoms in NiO_*x*_. Additionally, the XPS spectrum of the NiO_*x*_ film, shown in Figure 1g, reveals Ni 2p peaks at 854.49, 856.27, and 861.69 eV, corresponding to Ni^2+^, Ni^3+^, and the Ni^2+^ 2p satellite, respectively. The binding energy of Ni^3+^ in the NiO_*x*_/vitamin C film shifts to 855.93 eV, suggesting that vitamin C not only forms hydrogen bonds with the oxygen atoms of NiO_*x*_, but also coordinates with the nickel atoms (C = O···Ni^3+^). These findings indicate a strong interaction between vitamin C and NiO_*x*_. Figure 1h presents the XPS spectra of Pb 4f orbitals for PbI_2_ with and without vitamin C (1:1 molar ratio). The PbI_2_ spectrum shows 4f_7/2_ and 4f_5/2_ peaks at 138.38 and 143.27 eV, respectively, which shift to 137.87 and 142.74 eV in the PbI_2_:vitamin C mixture owing to electron donation from the C = O double bond in vitamin C, enhancing the electron density of Pb^2+^ ions. FTIR analysis is used to confirm the interaction between Pb^2+^ ions and the C = O groups of vitamin C by analyzing the C = O stretch (Figure 1i). The C = O stretch in vitamin C appears at 1756 cm^–1^, which shifts to 1748 cm^–1^ upon interaction with PbI_2_. These results validate the interactions of vitamin C with NiO_*x*_ (O–H···O, C = O···Ni^3+^) and with Pb^2+^ ions (C = O···Pb^2+^), as depicted in Figure 1j.

**Figure 2:**
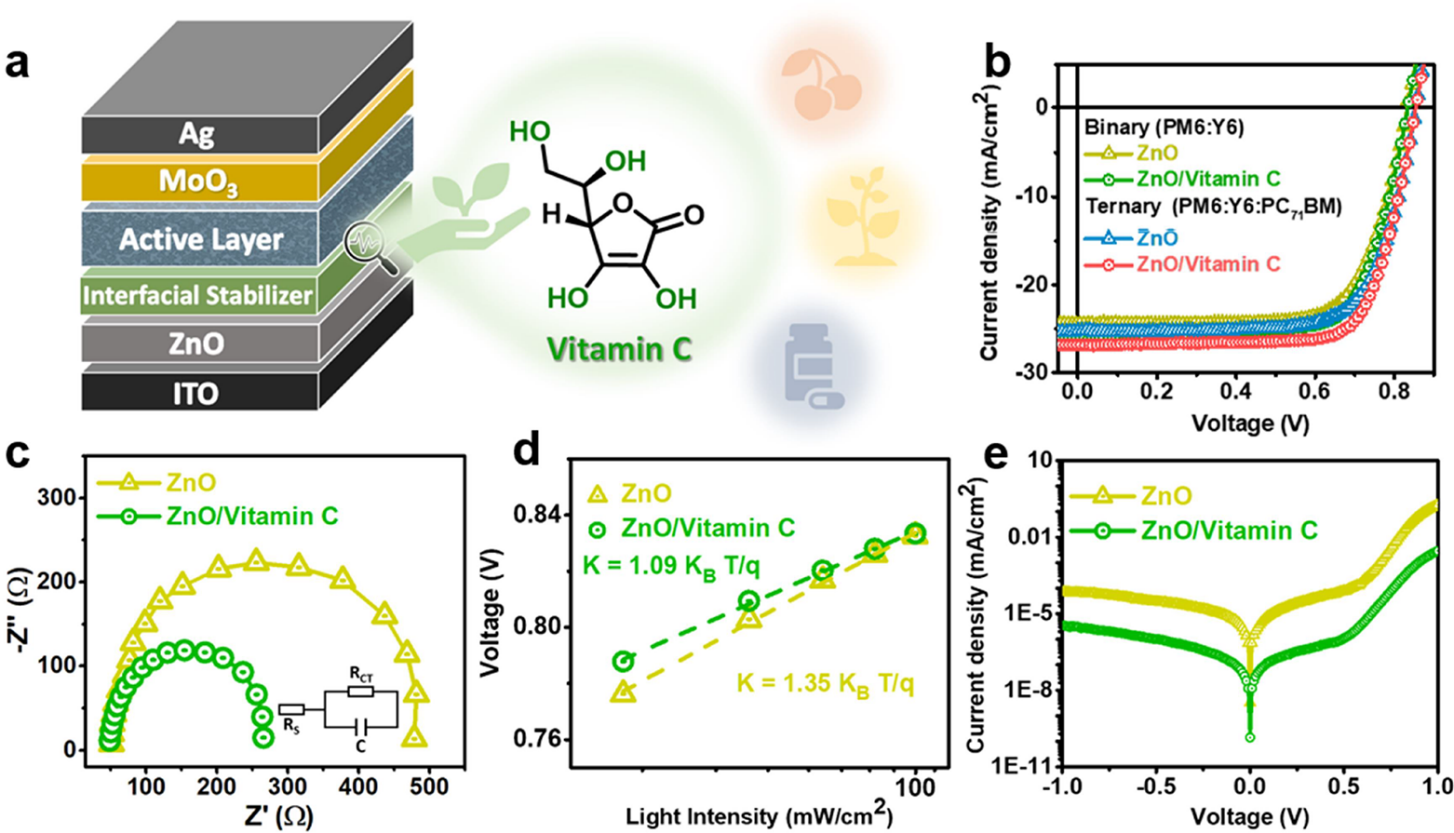
(a) Schematic diagram of the inverted OSC device structure, including chemical structure of vitamin C. (b) *J–V* characteristics of the inverted OSCs. (c) Nyquist plots, (d) *V*_*oc*_-light intensity relationship, and (e) *J–V* curves recorded under dark conditions for both ZnO and ZnO/vitamin C devices.

Figure 2a illustrates the device architecture for investigating the photovoltaic properties of ZnO and ZnO/vitamin C ETLs. Inverted devices were fabricated with the following structure: ITO/ZnO or ZnO/vitamin C ETLs/active layer/MoO_3_/Ag, where PM6:Y6 is employed as the active layer. Figure 2b depicts the *J–V* characteristics of the devices, while their photovoltaic parameters are listed in Table S1. The ZnO cell achieves a maximum PCE of 14.28% (short-circuit current density (*J*_*sc*_) = 24.39 mA/cm^2^, open-circuit voltage (*V*_*oc*_) = 0.832 V, fill factor (FF) = 70.37%), whereas the ZnO/vitamin C cell shows an 11% increase in PCE, reaching 15.78% (*J*_*sc*_ = 26.21 mA/cm^2^, *V*_*oc*_ = 0.834 V, FF = 72.18%). The ZnO/vitamin C ETL significantly enhances photovoltaic parameters, particularly FF and *J*_*sc*_. The external quantum efficiency (EQE) spectra, shown in Figure S1, demonstrate a broad photoresponse from 300 to 900 nm. The photocurrent densities calculated from the EQE spectra are 24.33 mA/cm^2^ for the ZnO device and 26.03 mA/cm^2^ for the ZnO/vitamin C device. The discrepancies between the EQE values and the *J*_*sc*_ values extracted from the *J–V* curves are 0.2% for the ZnO device and 0.3% for the ZnO/vitamin C device. The Nyquist plots from electrochemical impedance spectroscopy (EIS) measurements, along with the corresponding equivalent circuit model, are displayed in Figure 2c.^26^ The charge transport resistance (*R*_*CT*_) reflects the resistance at the electrodes/photoactive layer interfaces, while the series resistance (*R*_*S*_) represents the contact resistance at the layer interfaces and electrode contacts.^27−31^ The *R*_*CT*_ values for the ZnO and ZnO/vitamin C devices are 427.3 Ω and 217.1 Ω, respectively. The decreased *R*_*CT*_ in the ZnO/vitamin C device demonstrates that vitamin C effectively boosts charge transport in OSCs. The enhanced charge transport, leading to higher *J*_*sc*_ and lower *R*_*s*_, is attributed to vitamin C interactions with ZnO (O–H···O and C = O···Zn^2+^) and Y6 (O–H···F).

The defect density is investigated by determining the trap density (N_trap_) from the *I–V* responses in the space-charge-limited current (SCLC) regime.^32^ The calculated V_TFL_ values for the ZnO and ZnO/vitamin C devices are 0.61 and 0.45 V, respectively, which correspond to N_trap_ values of 2.38 × 10^16^ and 1.75 × 10^16^ cm^–3^, as shown in Figure S2. The devices’ response was measured, and the dependence of *V*_*oc*_ on light intensity was plotted.^26^ If trap-assisted recombination is absent, the slope should be 1 K_B_ T/q, but if traps are significant, the slope will exceed 1 K_B_ T/q.^33^ As shown in Figure 2d, the ZnO/vitamin C device has a slope of K = 1.09 K_B_ T/q, lower than the ZnO device’s slope of K = 1.35 K_B_ T/q, indicating reduced trap-assisted recombination. Figure 2e compares the dark currents of both devices, revealing that the ZnO/vitamin C device exhibits a lower dark current. These results indicate that the ZnO/vitamin C device suppresses charge recombination and improves the photovoltaic parameters, specifically *R*_*sh*_.

Ternary systems have attracted significant attention in recent years as an effective strategy for improving the performance of OSCs.^34−37^ Therefore, this study fabricated ternary solar cell devices (PM6:Y6:PC_71_BM) by adding an extra PC_71_BM acceptor to the PM6:Y6 bulk heterojunction (BHJ) and utilizing ZnO/vitamin C as the ETL.^38−40^ The *J–V* curves of the ternary devices are presented in Figure 2b, with their corresponding photovoltaic parameters summarized in Table S1. The maximum PCE of ternary devices using ZnO is 16.04% (*J*_*sc*_ = 25.29 mA/cm^2^, *V*_*oc*_ = 0.852 V, and FF = 74.44%), while the highest PCE increases by 10% to 17.66% (*J*_*sc*_ = 26.85 mA/cm^2^, *V*_*oc*_ = 0.857 V, and FF = 76.74%) with the ZnO/vitamin C ETL. The EQE-derived photocurrent densities for the ZnO and ZnO/vitamin C devices are 25.27 and 26.58 mA/cm^2^, with discrepancies of 0.07% and 0.5% compared to the *J*_*sc*_ values from the *J–V* curves (Figure S1). Ultimately, the interaction between vitamin C and both ZnO and Y6 can simultaneously improve charge extraction efficiency in binary and ternary systems, reduce charge recombination, and enhance interface compatibility.

**Figure 3:**
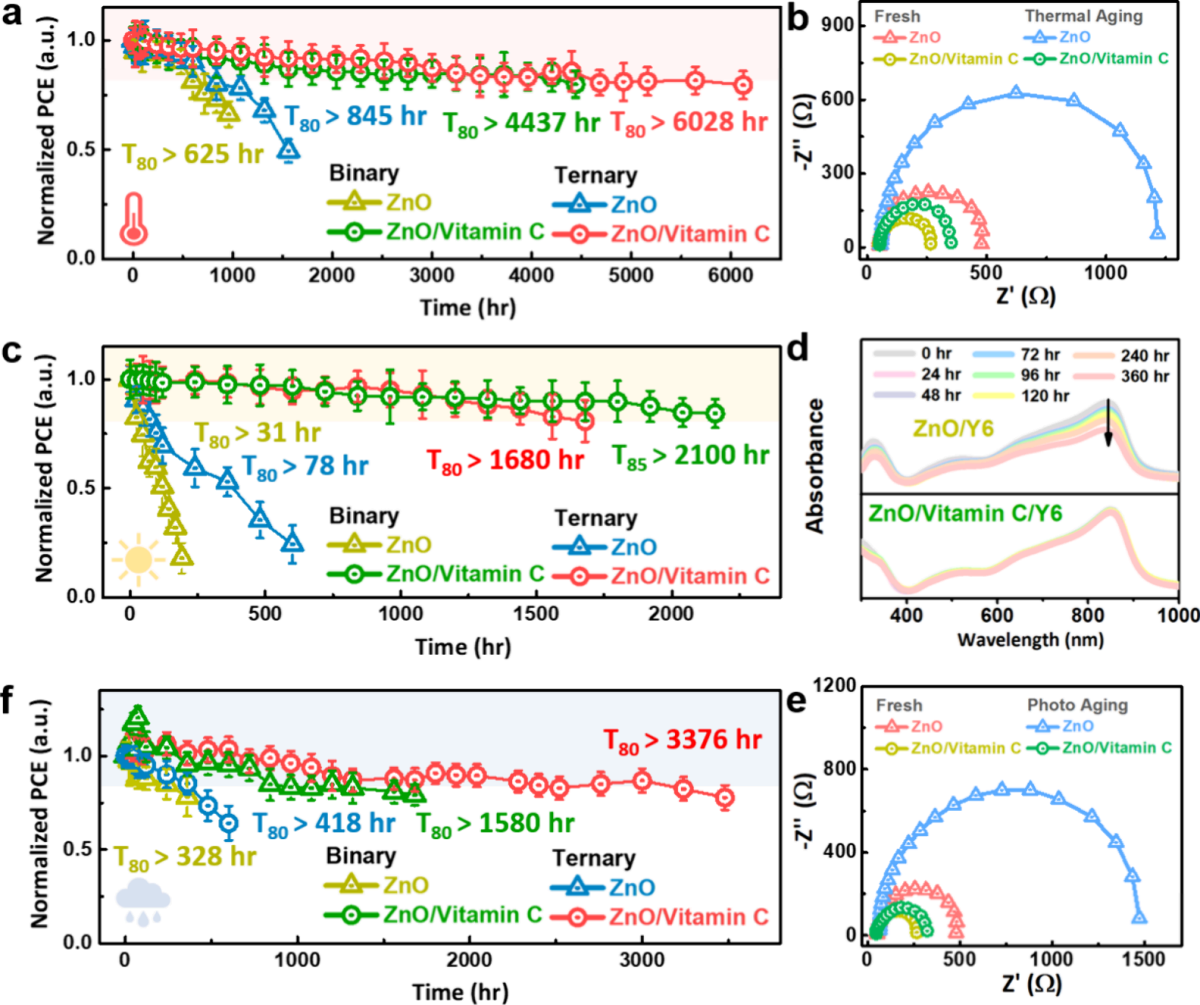
(a) Thermal stability of devices at 65 °C in N_2_ atmosphere. (b) Nyquist plots of devices after 800 h of thermal aging at 65 °C in a N_2_ environment, compared to fresh devices. (c) Photostability of devices under continuous one-sun illumination with an AM1.5G filter in a N_2_ atmosphere. (d) UV–vis absorption spectra of ZnO/Y6 and ZnO/vitamin C/Y6 samples under continuous one-sun illumination with an AM1.5G filter in a N_2_ atmosphere, measured at various time intervals. (e) Nyquist plots of devices after 120 h of photoaging under AM1.5G simulated sunlight in a N_2_ atmosphere, compared to fresh devices. (f) Humidity stability of devices under 50% relative humidity at 25 °C.

The TGA curve in Figure S3 demonstrates that vitamin C exhibits excellent thermal stability with a high *T*_*d, 95%*_ of 234 °C. To evaluate the thermal stability of our binary devices, we stored them at 65 °C in an N_2_ atmosphere. The ZnO/vitamin C device maintains a T_80_ lifetime of 4,437 h, significantly outperforming the ZnO device, which has a lifetime of only 625 h, as shown in Figure 3a. These results demonstrate the superior thermal stability of the ZnO/vitamin C device. The origin of thermal degradation in the devices was investigated by analyzing the evolution of their photovoltaic characteristics, as depicted in Figure S4. Notably, the rapid performance decline is primarily due to a reduction in FF, while *J*_*sc*_ and *V*_*oc*_ are only minimally impacted. The devices were stored under N_2_ and subjected to annealing at 65 °C for 800 h to evaluate the effects of thermal aging on the interface. As shown in Figure S5a, the trap density of the ZnO device rose markedly from 2.38 × 10^16^ to 2.69 × 10^16^ cm^–3^, whereas that of the ZnO/vitamin C device only slightly increased from 1.75 × 10^16^ to 1.95 × 10^16^ cm^–3^ (summarized in Table S2). By analyzing the relationship between *V*_*oc*_ and illumination intensity, we found that the ZnO/vitamin C device showed a smaller increase in trap-assisted recombination, with its slope varying from K = 1.09 K_B_ T/q to 1.15 K_B_ T/q. In contrast, the ZnO device exhibited a slope of K = 1.48 K_B_ T/q, which was higher than its initial value of K = 1.35 K_B_ T/q (Figure S5b, Table S2).^33^ After thermal aging, the trap-assisted recombination of the ZnO device increases, leading to a significant decrease in *R*_*sh*_. As shown in Figure 3b, electrochemical impedance measurements revealed a significant increase in the *R*_*CT*_ value for the ZnO device, from 427.3 to 1161.1 Ω, while the ZnO/vitamin C device experienced a smaller rise, from 217.1 to 299.9 Ω. The substantial increase in *R*_*s*_ for the ZnO device during thermal aging was primarily attributed to a significant rise in *R*_*CT*_, reflecting critical degradation of charge transport at the inorganic/organic interface. These results highlight that incorporating vitamin C improves the stability of inverted OSCs, with the PCE reduction mainly ascribed to interface degradation. The test conducts at 65 °C primarily focuses on the interfacial phenomenon rather than BHJ changes, which require higher temperatures. Furthermore, as shown in Figure 3a, the ZnO/vitamin C device in the ternary system demonstrates excellent thermal stability, maintaining its T_80_ lifetime of 6,028 h at 65 °C in an N_2_ atmosphere. In contrast, the ZnO device in the ternary system shows a T_80_ lifetime of only 845 h under the same conditions. Figure S6 illustrates the evolution of all device parameters for the ternary devices during thermal aging at 65 °C in a nitrogen environment, showing trends consistent with those of the binary devices. This enhancement is due to vitamin C’s interactions with ZnO (via O–H···O and C = O···Zn^2+^ bonds) and Y6 (via O–H···F bonds), which create a strong interface that can withstand high-temperature stress, thereby improving the stability of OSCs.

We assessed the photostability of the binary devices by exposing them to continuous one-sun illumination with an AM1.5G filter in an N_2_ condition. Figure 3c shows a significant decrease in the PCE of the ZnO device, with its T_80_ lifetime limited to just 31 h. In contrast, the ZnO/vitamin C device retains 85% of its initial PCE after 2,100 h. Figure S7 presents the normalized parameter changes of the binary OSCs during photoaging. Further analysis reveals that the FF and *J*_*sc*_ of the ZnO device contribute most to performance degradation, while *V*_*oc*_ only slightly decreases. ZnO’s intrinsic photocatalytic activity generates reactive radicals like •OH and •O^2–^ under UV light, which break double bonds between electron-rich and electron-deficient units in the NFA structure.^11−14^Figure 3d shows the absorption spectra evolution of ZnO/Y6 and ZnO/vitamin C/Y6 samples under continuous illumination with AM1.5G simulated sunlight at one-sun intensity in an N_2_ environment. The ZnO/Y6 sample exhibited a significant decrease in absorbance, while the ZnO/vitamin C/Y6 sample showed only a minor reduction. This result provides clear evidence of the effectiveness of vitamin C in preventing the photocatalytic degradation of Y6 on ZnO, likely due to its free radical scavenging ability and its role in blocking direct contact between Y6 and ZnO. Additionally, we conducted an analysis after photoaging, which involved one-sun illumination for 120 h under N_2_ conditions. As shown in Figure 3e, electrochemical impedance analysis revealed that the *R*_*CT*_ value of the ZnO device rose significantly from 427.3 to 1409.1 Ω, while the ZnO/vitamin C device exhibited a smaller increase, from 217.1 to 275.8 Ω. This suggests that vitamin C helps preserve *R*_*CT*_ after photoaging, supporting the stability of *R*_*s*_ and *J*_*sc*_. Figure S8a shows that the trap density of the ZnO device grew notably from 2.38 × 10^16^ to 2.57 × 10^16^ cm^–3^, whereas the ZnO/vitamin C device saw a minor increase from 1.75 × 10^16^ to 1.79 × 10^16^ cm^–3^ (summarized in Table S3). The *V*_*o*c_-illumination intensity analysis reveals a greater rise in trap-assisted recombination for the ZnO device, with K rising from 1.35 K_B_ T/q to 1.45 K_B_ T/q. In contrast, the ZnO/vitamin C device showed minimal change, with K going from 1.09 K_B_ T/q to 1.11 K_B_ T/q (as shown in Figure S8b and summarized in Table S3). These results suggest that vitamin C forms a robust interface with both ZnO and Y6, effectively reducing trap-assisted recombination and maintaining *R*_*sh*_ stability. We also examine the ternary devices by storing them under one-sun illumination with an AM1.5G filter in an N_2_ condition. As illustrated in Figure 3c, the ZnO/vitamin C device in the ternary system still retains its T_80_ lifetime after more than 1,680 h of photoaging. In contrast, the PCE of the ZnO device degraded rapidly, with a T_80_ lifetime of only 78 h. Figure S9 presents the variations in all device parameters for the ternary devices under one-sun illumination with an AM1.5G filter in a nitrogen environment, revealing trends consistent with those observed in the binary devices. To evaluate the humidity stability of binary and ternary systems, we performed tests on unencapsulated ZnO and ZnO/vitamin C devices under the relative humidity of 50% at 25 °C. As illustrated in Figure 3f, the ZnO/vitamin C device in the binary system achieved a T_80_ lifetime of 1,580 h, significantly surpassing the 328 h of the ZnO device. In the ternary system, the T_80_ lifetime of ZnO devices is 418 h, while the ZnO/vitamin C device can maintain its functionality even after being exposed to moisture for more than 3,376 h. The passivation of surface defects and dangling bonds contributes to the enhanced humidity stability of ZnO/vitamin C devices.

**Figure 4:**
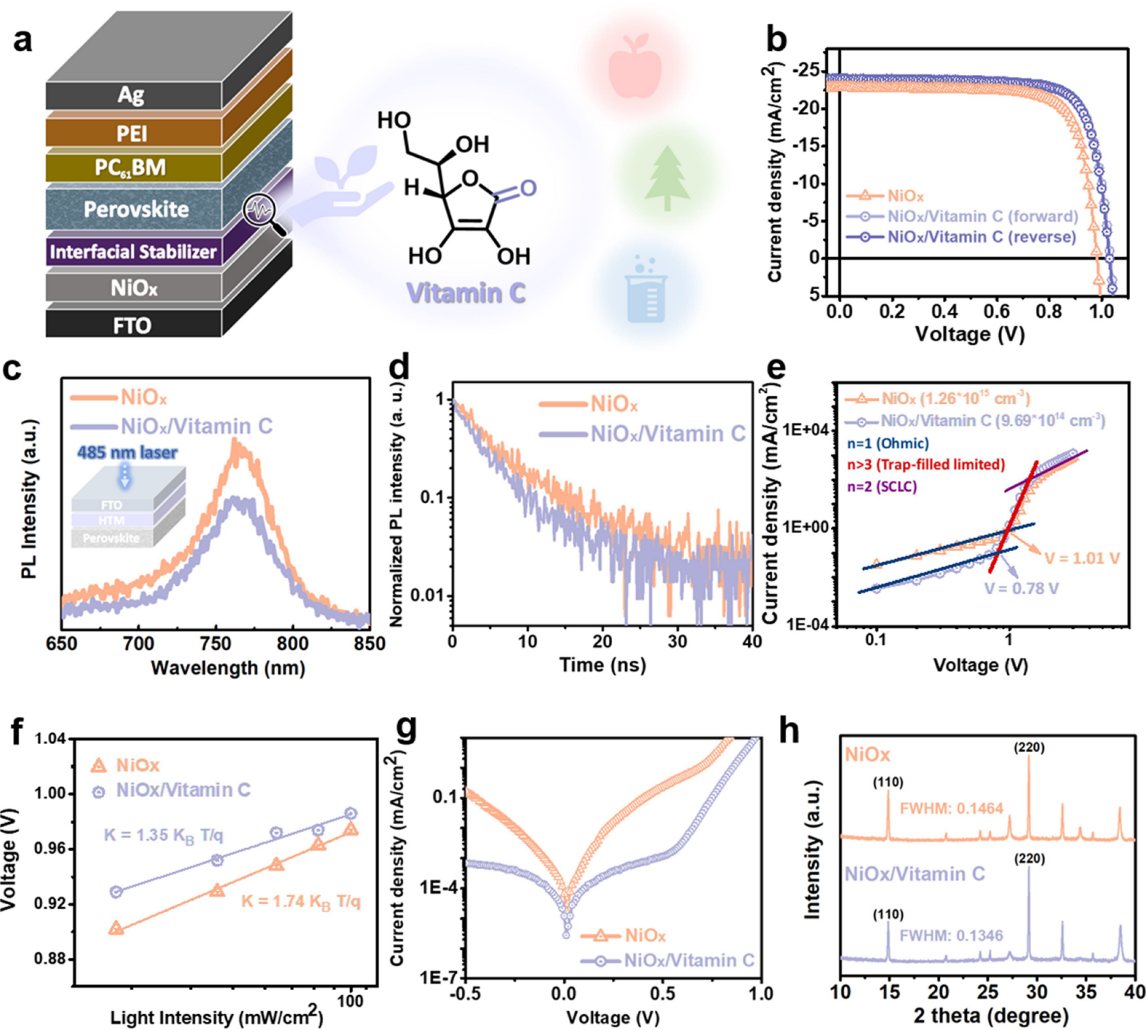
(a) Schematic illustration of the inverted PSC device structure alongside the chemical structure of vitamin C. (b) *J–V* characteristics of inverted PSCs. (c) Steady-state PL spectra and (d) time-resolved PL spectra of NiO_*x*_/perovskite and NiO_*x*_/vitamin C/perovskite samples. For devices with NiO_*x*_ and NiO_*x*_/vitamin C HTMs, the following measurements were performed: (e) trap density, (f) *V*_*oc*_ dependence on light intensity, and (g) dark *J–V* characteristics. (h) XRD patterns showing the crystallinity of perovskite layers on NiO_*x*_ and NiO_*x*_/vitamin C HTMs.

In addition to the aforementioned studies, vitamin C has also been applied in inverted PSCs in combination with NiO_*x*_, forming a NiO_*x*_/vitamin C layer as an HTM. A p-i-n device is created using an inverted architecture of either fluorine-doped tin oxide (FTO)/NiO_*x*_ or NiO_*x*_/vitamin C HTMs/perovskite layer/PC_61_BM/polyethylenimine/Ag, as illustrated in Figure 4a. Figure 4b presents the *J–V* curves of the devices, with their photovoltaic parameters summarized in Table S4. The champion PCE of the device with NiO_*x*_ is 17.47% (*J*_*sc*_ = 22.89 mA/cm^–2^, *V*_*oc*_ = 1.00 V, and FF = 76.30%), whereas that of the NiO_*x*_/vitamin C device achieves a 9% to 19.10% (*J*_*sc*_ = 23.90 mA/cm^–2^, *V*_*oc*_ = 1.03 V, and FF = 77.31%). The NiO_*x*_/vitamin C HTM markedly enhances device performance, especially *J*_*sc*_ and FF. Figure S10 presents the EQE spectra of the devices, showing integrated photocurrents of 22.62 and 23.57 mA/cm^2^ for the NiO_*x*_ and NiO_*x*_/vitamin C devices, respectively. The discrepancy between the EQE values of the NiO_*x*_ and NiO_*x*_/vitamin C devices and the *J*_*sc*_ values obtained from the *J–V* measurements is 0.2% and 0.3%, respectively. To assess interfacial charge transport, we perform photoluminescence (PL) measurements of NiO_*x*_/perovskite and NiO_*x*_/vitamin C/perovskite samples using 485 nm excitation. As shown in Figure 4c, the emission from the perovskite is partially quenched by the NiO_*x*_/vitamin C HTM, suggesting that NiO_*x*_/vitamin C facilitates charge extraction at the interface. Additionally, the time-resolved photoluminescence (TRPL) spectra of perovskite films are measured for both NiO_*x*_ and NiO_*x*_/vitamin C HTMs, and the data are fitted with a sum of two exponential decay functions, as shown in Figure 4d. The fast decay component τ_1_ is related to interfacial charge separation properties, while the slow decay component τ_2_ is associated with nonradiative recombination properties in bulk.^41−43^ The value of τ_1_ for NiO_*x*_/vitamin C (2.44 ns) is shorter than that of NiO_*x*_ (4.58 ns), with a summary of the data presented in Table S5. The improved charge extraction efficiency, leading to increased *J*_*sc*_ and reduced *R*_*s*_, is attributed to the interactions between vitamin C and NiO_*x*_ (O–H···O and C = O···Ni^3+^) as well as with the perovskite layers (C = O···Pb^2+^).

To better understand the defect density, the trap density in the devices was evaluated through *I–V* analysis in the SCLC regime. As shown in Figure 4e, the NiO_*x*_/vitamin C device exhibited a lower V_TFL_ of 0.78 V compared to 1.01 V for the NiO_*x*_ device, resulting in N_trap_ of 9.69 × 10^14^ cm^–3^ and 1.26 × 10^15^ cm^–3^, respectively. To further explore carrier recombination during device operation, we analyzed the *V*_*oc*_ dependence on light intensity. As shown in Figure 4f, the NiO_*x*_/vitamin C device (K = 1.35 K_B_ T/q) has a significantly lower slope compared to the NiO_*x*_ device (K = 1.74 K_B_ T/q), indicating reduced trap-assisted recombination. Figure 4g reveals that the NiO_*x*_/vitamin C device exhibits lower dark current, suggesting that its HTM effectively suppresses charge recombination. X-ray diffraction (XRD) analysis shows identical crystallinity peaks for the perovskite layers on both NiO_*x*_ and NiO_*x*_/vitamin C HTMs (Figure 4h). The crystal sizes, estimated by fitting the full-width-at-half-maximum of the (110) peak, are 56.08 nm for NiO_*x*_ and 61.03 nm for NiO_*x*_/vitamin C. Scanning electron microscopy (SEM) images reveal that perovskite films on NiO_*x*_/vitamin C HTMs have larger crystal grains (306 nm) compared to those on NiO_*x*_ (223 nm), as shown in Figure S11a-c. Furthermore, the TRPL spectra in Figure S12 show that the perovskite film on NiO_*x*_/vitamin C HTLs exhibits a significantly longer exciton lifetime. These results indicate that NiO_*x*_/vitamin C HTM enhances perovskite film quality by effectively passivating the surface defects of NiO_*x*_, leading to improvements in *J*_*sc*_, *V*_*oc*_, and *R*_*sh*_.

**Figure 5:**
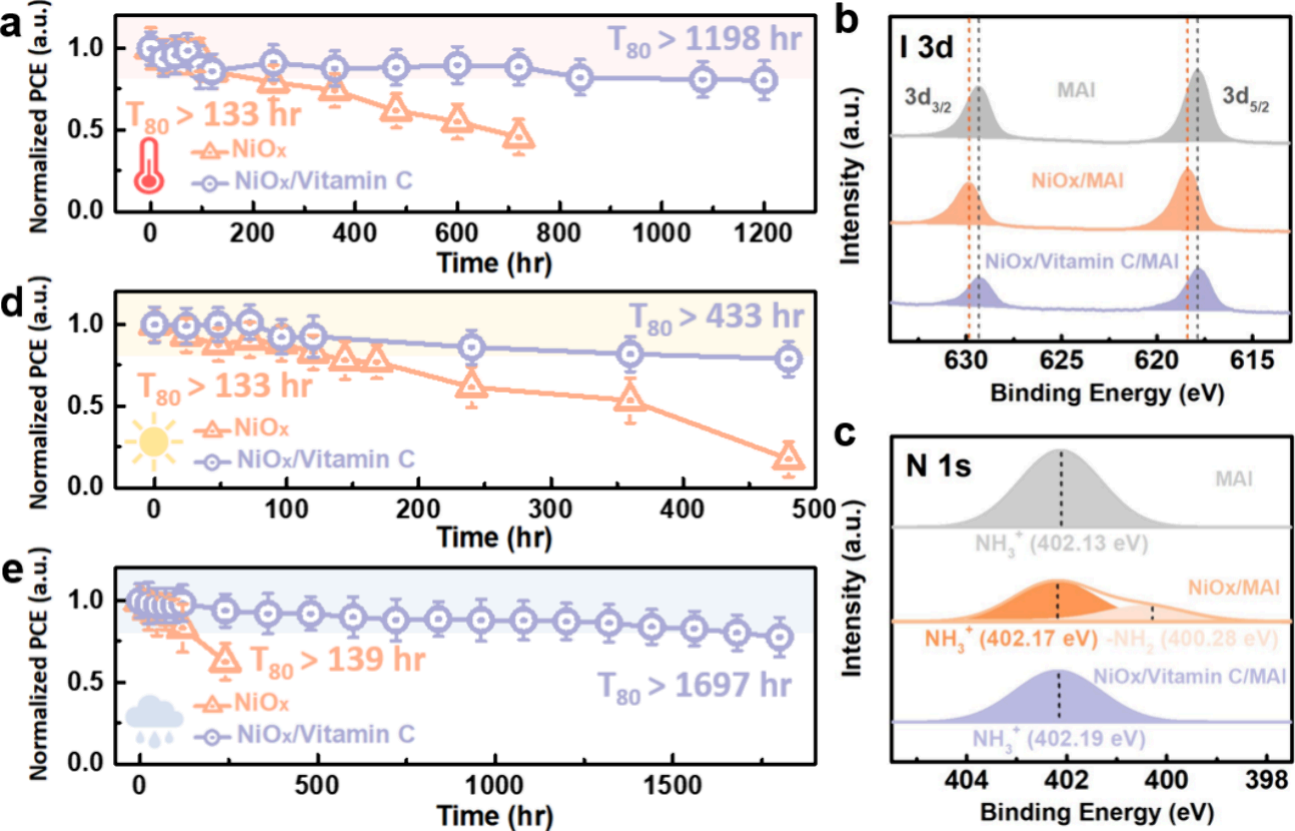
(a) Thermal stability of the devices at 65 °C in a N_2_ atmosphere. XPS analysis of (b) I 3d and (c) N 1s for pristine MAI powder, as well as annealed NiO_*x*_/MAI and MAI/vitamin C/NiO_*x*_ samples. (d) Photostability of the devices under AM1.5G one-sun illumination in a N_2_ atmosphere. (e) Humidity stability of the devices at 50% relative humidity and 25 °C.

We evaluated the effect of vitamin C on the stability of PSCs through continuous thermal stress at 65 °C in an N_2_ environment. The NiO_*x*_/vitamin C device achieves a T_80_ lifetime of 1,198 h, significantly outperforming the NiO_*x*_ device, which has a T_80_ lifetime of only 218 h, as shown in Figure 5a. To further explore the chemical interactions among the NiO_*x*_ HTM, I^–^ ions, and Ni^3+^ ions at the interface, we deposited thin layers of methylammonium iodide (MAI) on various substrates at 65 °C in an N_2_ environment for 12 h to observe any changes in the films. After thermal annealing, the MAI layers on both FTO and NiO_*x*_/vitamin C substrates retained transparency and showed no visible color change, as shown in Figure S13. In contrast, the MAI on the NiO_*x*_ substrate underwent a chemical transformation, evidenced by the appearance of a brown color. In Figure 5b, the XPS spectrum of MAI powder shows I 3d peaks at 617.85 and 529.35 eV, attributed to the 3d_5/2_ and 3d_3/2_ orbitals. In the annealed MAI/NiO_*x*_ sample, these peaks shift to higher binding energies at 618.38 and 629.86 eV. This shift suggests that the I^–^ ions in the annealed NiO_*x*_/MAI sample underwent oxidation due to interaction with Ni^3+^. In contrast, no such shift was observed in the annealed MAI/vitamin C/NiO_*x*_ sample, indicating no reaction at the interface. Figure 5c shows the N 1s signal at 402.13 eV in the XPS spectrum of MAI powder, attributed to NH_3_^+^ species. Among the samples, the annealed MAI/NiO_*x*_ sample exhibited the most pronounced deprotonation after thermal annealing, as indicated by the -NH_2_ peak at 400.28 eV. Meanwhile, the annealed MAI/vitamin C/NiO_*x*_ sample showed no deprotonation peaks, suggesting that vitamin C prevents the deprotonation of MAI. This protective effect likely arises from coordination between vitamin C and Ni^3+^ atoms (C = O···Ni^3+^), reducing the likelihood of its reaction with MAI. Additionally, we evaluated device stability under light exposure and humidity. Figure 5d shows that the NiO_*x*_/vitamin C device achieves a T_80_ lifetime of 433 h under one-sun illumination with an AM1.5G filter, which is considerably longer than the 133-h T_80_ lifetime of the NiO_*x*_ device. As shown in Figure 5e, the unencapsulated NiO_*x*_ and NiO_*x*_/vitamin C devices were tested at 50% relative humidity and 25 °C. The NiO_*x*_/vitamin C device achieved a T_80_ lifetime of 1,697 h, far surpassing the NiO_*x*_ device’s 139 h. The NiO_*x*_/vitamin C device exhibits superior stability against heat, light, and humidity, attributed to enhanced perovskite quality and stronger interfacial interactions that mitigate degradation.

## Conclusions

This study investigates the underlying mechanism through which vitamin C functions as a universal interfacial stabilizer. In OSCs, XPS and FTIR analyses uncover that vitamin C interacts with ZnO (O–H···O and C = O···Zn^2+^) and Y6 (O–H···F), thereby enhancing interfacial stability. Our results show that both binary and ternary systems incorporating ZnO/vitamin C ETLs achieve T_80_ lifetimes of 4,437 and 6,028 h, respectively, under storage at 65 °C in a nitrogen atmosphere. Under one-sun illumination with an AM1.5G filter in a nitrogen environment, these devices exhibit exceptional stability, with the binary device retaining 85% of its original PCE for 2,100 h and the ternary device achieving a T_80_ lifetime of 1,680 h. In PSCs, vitamin C stabilizes the interface between NiO_*x*_ (O–H···O and C = O···Ni^3+^) and the perovskite layer (C = O···Pb^2+^). The NiO_*x*_/vitamin C HTM-based device exhibits a T_80_ lifetime of 1,198 h under thermal stress at 65 °C in a nitrogen atmosphere. These findings provide effective strategies for improving the practicality and durability of photovoltaic devices.

## Experimental Section

### Device Fabrication

Inverted OSCs (ITO glass/ZnO or ZnO/vitamin C/active layer/MoO_3_/Ag)^26^ and inverted PSCs (FTO glass/NiO_*x*_ or NiO_*x*_/vitamin C/active layer/PC_61_BM/polyethylenimine/Ag)^44^ were fabricated according to previously reported procedures. The active areas of the OSCs and PSCs were 0.067 cm^2^ and 0.038 cm^2^, respectively. A 0.1 mg/mL solution of vitamin C in MeOH was spin-coated onto the ITO/ZnO ETL or FTO/NiO_*x*_ HTM substrates at 4000 rpm for 30 s, followed by annealing at 110 °C for 30 s in an N_2_ glovebox. The resulting vitamin C layer had a thickness of approximately 1–2 nm, as measured using a surface profiler (α-stepper).

### Stability Testing

Ten devices were tested under each condition, and error bars were calculated for statistical accuracy. Thermal stability was evaluated through prolonged heating in an N_2_ environment. Light stability was tested under continuous AM1.5G illumination using a Newport LSH-7320 solar simulator. Humidity stability was evaluated using a TEN-H40 environmental chamber (Tender Scientific) under controlled conditions, with temperatures ranging from 0 to 100 °C (±1 °C) and humidity levels between 25% and 95%.

## Supporting Information Available

Detailed fabrication process and characterization of OSCs and PSCs in this study; EQE spectra of all devices; current–voltage traces and trap density; evolution of normalized parameters of the studied OSCs under thermal aging or photoaging; TGA curve of Vitamin C; SEM images (PDF)
