# Supplementary material for: Insights into the Role of Vitamin C in Stabilizing Organic and Perovskite Solar Cells
Source: ACS Appl Mater Interfaces. 2025 Feb 18;17(8):12339–47. doi: 10.1021/acsami.4c22532 (PMC11873947; doi:10.1021/acsami.4c22532)
Supplement: Supplementary file 1 — am4c22532_si_001.pdf [file am4c22532_si_001.pdf]

Supporting Information

Insights into the Role of Vitamin C in Stabilizing Organic and Perovskite Solar Cells

Guan-Lin Chen,[a, b, c] Kai-Wei Tseng,[a, b, c] Ching-I Huang,[a, *] Leeyih Wang,[a, b, c, *]

[a] Institute of Polymer Science and Engineering, National Taiwan University, Taipei, 10617, Taiwan

[b] Center for Condensed Matter Sciences, National Taiwan University, Taipei, 10617, Taiwan

[c] Center of Atomic Initiative for New Materials, National Taiwan University, Taipei 10617, Taiwan

* Corresponding authors' email addresses: chingih@ntu.edu.tw, leewang@ntu.edu.tw

KEYWORDS: *Vitamin C, eco-friendly, interfacial interaction, robust interface, stability*

Table S1 Photovoltaic performance of the inverted OSCs devices based on the ZnO and ZnO/vitamin C ETLs for the binary and ternary systems.

Active Layer	Parameters	V_{oc} (V)	J_{sc} (mA/cm^2)	Fill factor (%)	PCE (%)	R_{sh}(kΩ*cm^2)	R_s(Ω*cm^2)
Binary	ZnO	0.830±0.002 (0.832)	24.28±0.10 (24.39)	70.20±0.11 (70.37)	14.19±0.09[a] **(14.28)**[b]	0.69±0.14 (0.86)	3.12±0.13 (2.96)
	ZnO/Vitamin C	0.830±0.004 (0.834)	25.56±0.25 (26.21)	71.93±0.39 (72.18)	15.26±0.33[a] **(15.78)**[b]	0.91±0.16 (1.09)	2.72±0.19 (2.66)
Ternary	ZnO	0.850±0.001 (0.852)	25.04±0.21 (25.29)	74.23±0.16 (74.44)	15.80±0.21[a] **(16.04)**[b]	1.33±0.12 (1.48)	3.79±0.07 (3.57)
	ZnO/Vitamin C	0.856±0.001 (0.857)	25.98±0.14 (26.85)	75.54±0.12 (76.74)	16.80±0.15[a] **(17.66)**[b]	1.82±0.11 (2.14)	3.43±0.18 (3.23)

a. The average PCEs are based on 15 devices. b. Parentheses show the values from the champion devices.

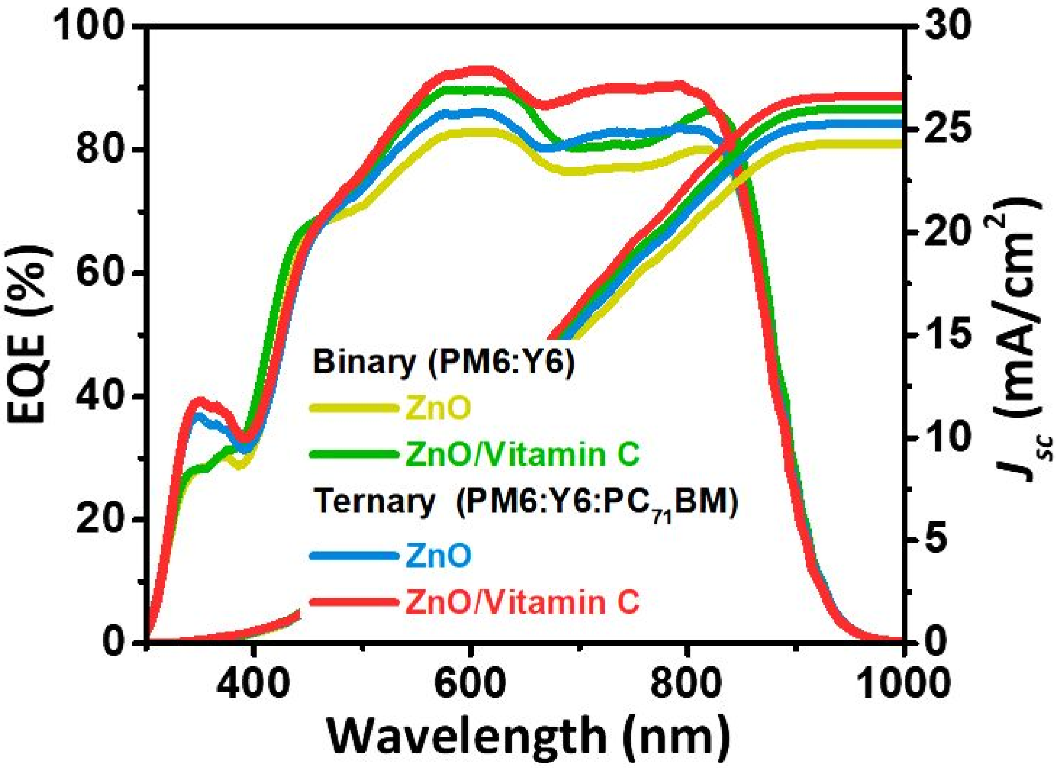

Figure S1. EQE spectra of our fabricated inverted OSCs using the ZnO and ZnO/vitamin C ETLs.

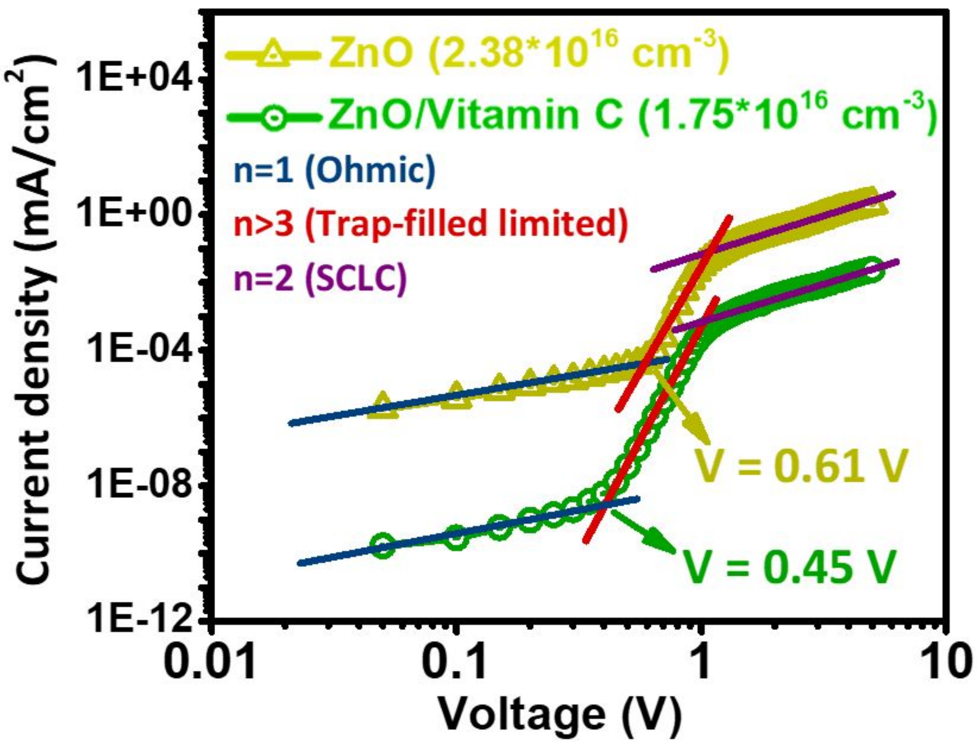

Figure S2. Current-voltage characteristics and trap density of ZnO and ZnO/vitamin C devices

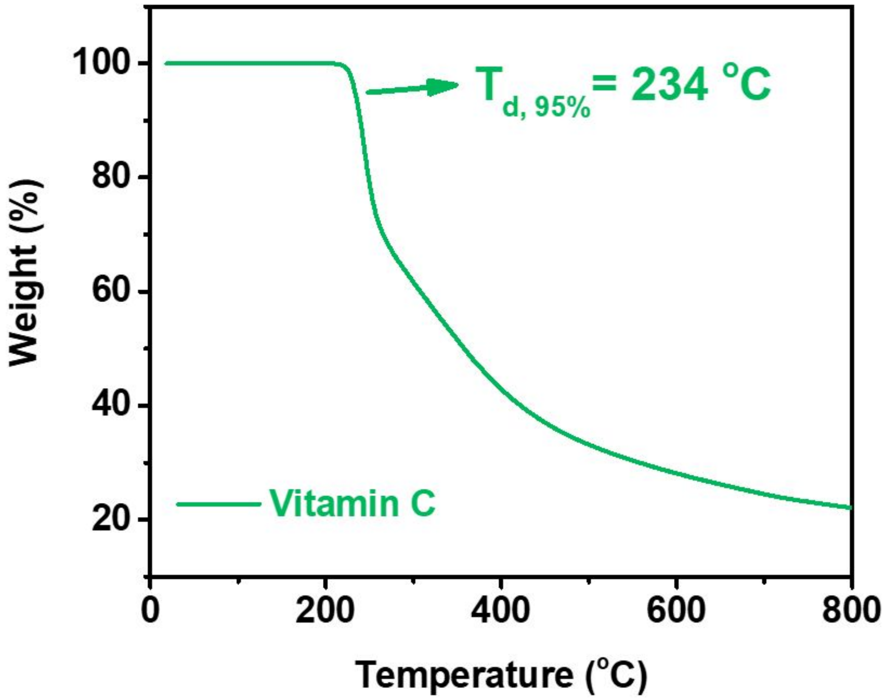

Figure S3. TGA curve of Vitamin C.

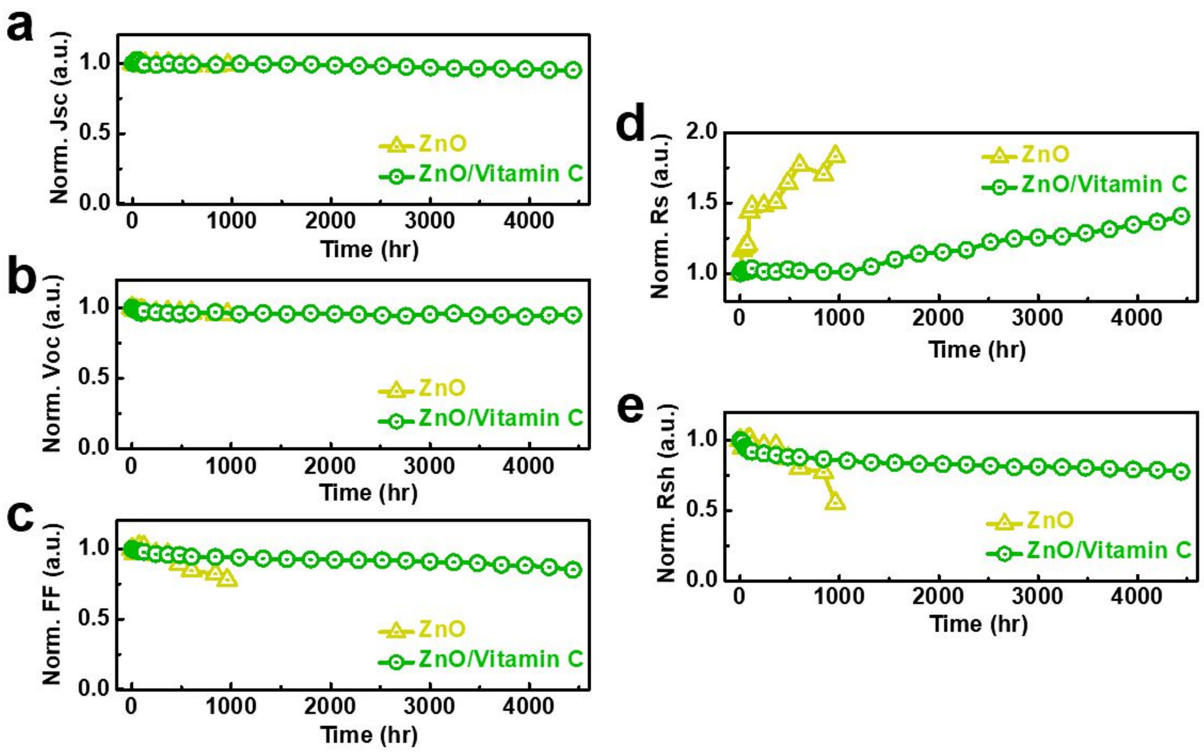

Figure S4. The evolutions of normalized (a) J_{sc}, (b) V_{oc}, (c) FF, (d) R_s, and (e) R_{sh} of the binary OSCs under thermal aging at 65 °C in N_2.

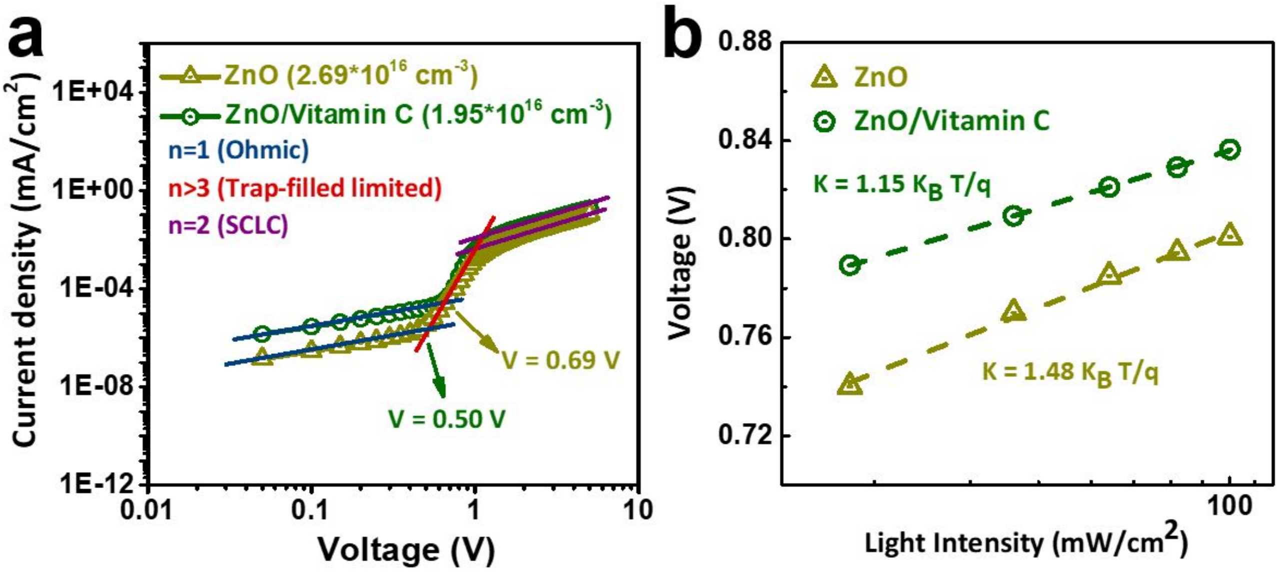

Figure S5. (a) Current-voltage traces and trap density of ZnO and ZnO/vitamin C. (b) V_{oc} as a function of light intensity of ZnO and ZnO/vitamin C devices. These devices were thermally annealed at 65 °C for 800 hr in N_2 prior measurement.

Table S2 Compared with the fresh and photo aging devices with trap density and the slope (K) of the relationship between V_{oc} and illumination intensity for the ZnO and ZnO/vitamin C devices.

	Fresh		Thermal Aging	
**Parameters**	N_{trap}	K (K_B T/q)	N_{trap}	K (K_B T/q)
**ZnO**	$2.38*10^{16}$ cm^{-3}	1.35	$2.69*10^{16}$ cm^{-3}	1.48
**ZnO/Vitamin C**	$1.75*10^{16}$ cm^{-3}	1.09	$1.95*10^{16}$ cm^{-3}	1.15

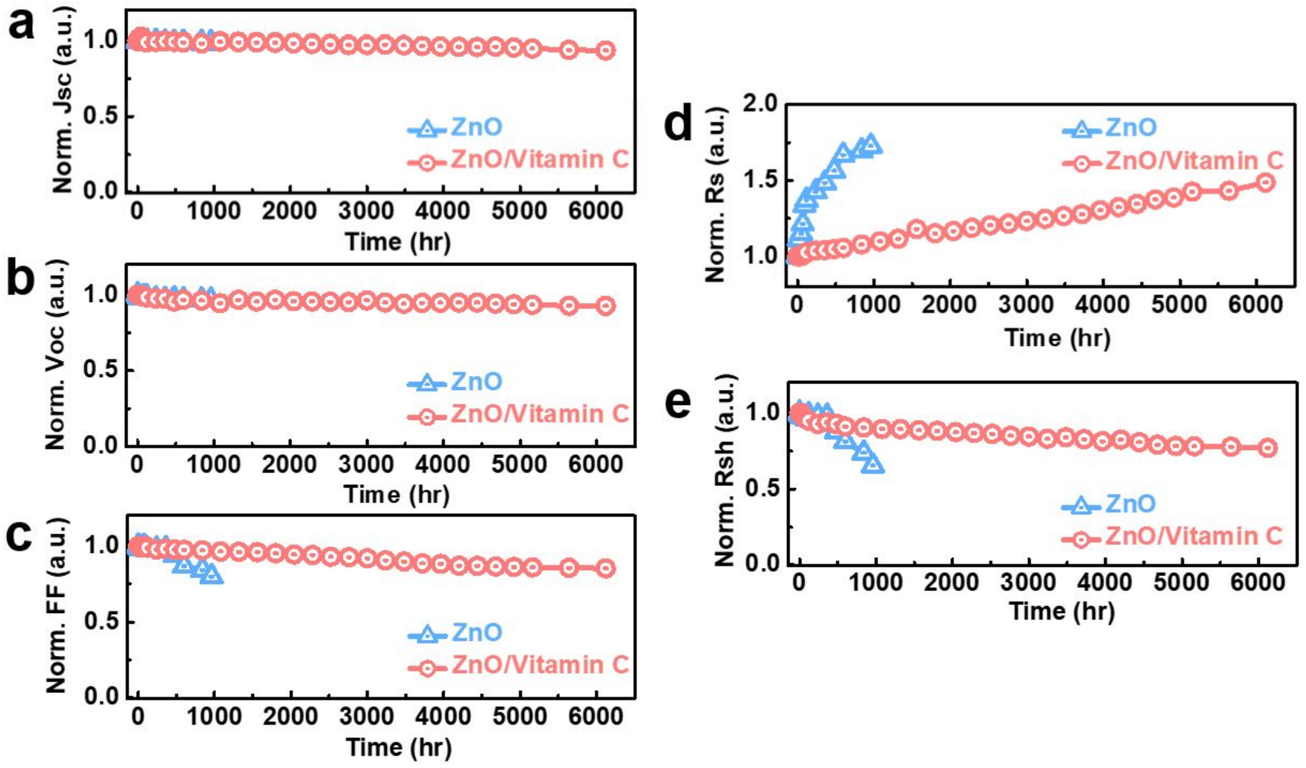

Figure S6. The evolutions of normalized (a) J_{sc}, (b) V_{oc}, (c) FF, (d) R_s, and (e) R_{sh} of the ternary OSCs under thermal aging at 65 °C in N_2.

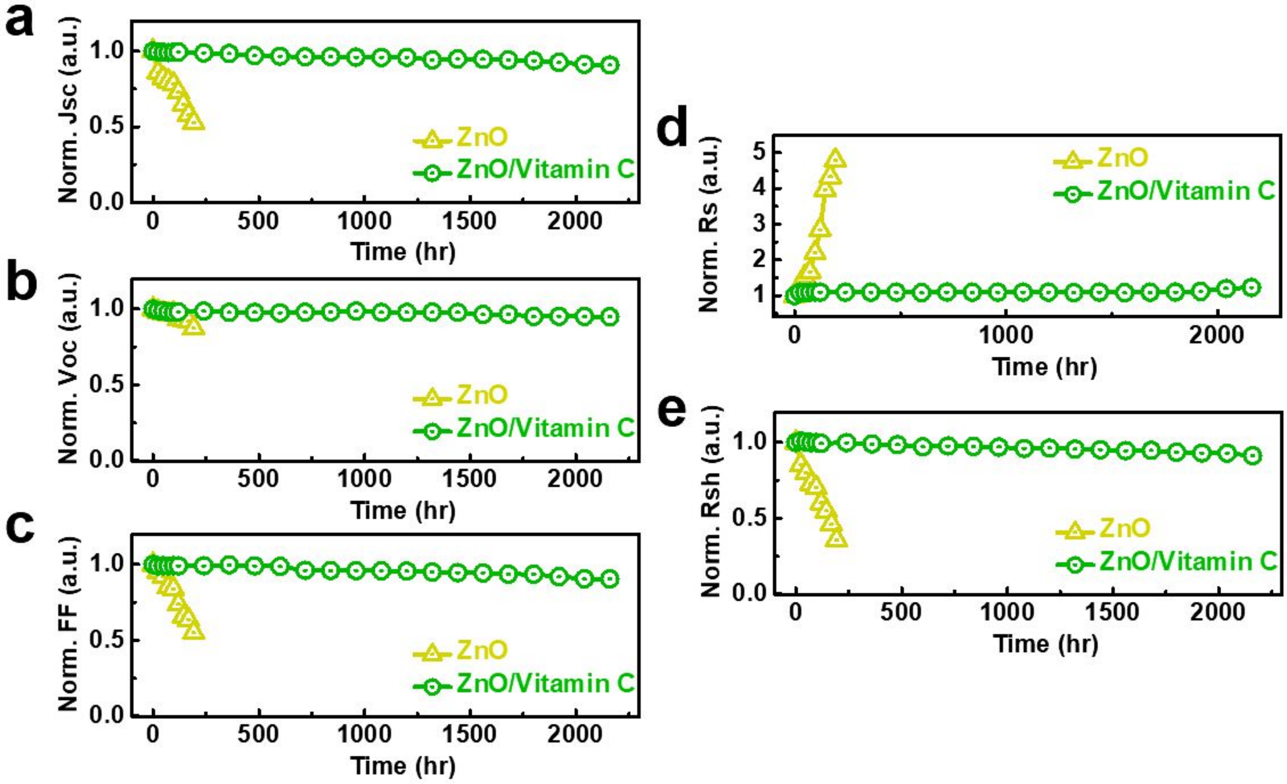

Figure S7. The evolutions of normalized (a) J_{sc}, (b) V_{oc}, (c) FF, (d) R_s, and (e) R_{sh} of the binary OSCs under continuous one-sun illumination with an AM1.5G filter in N_2 condition.

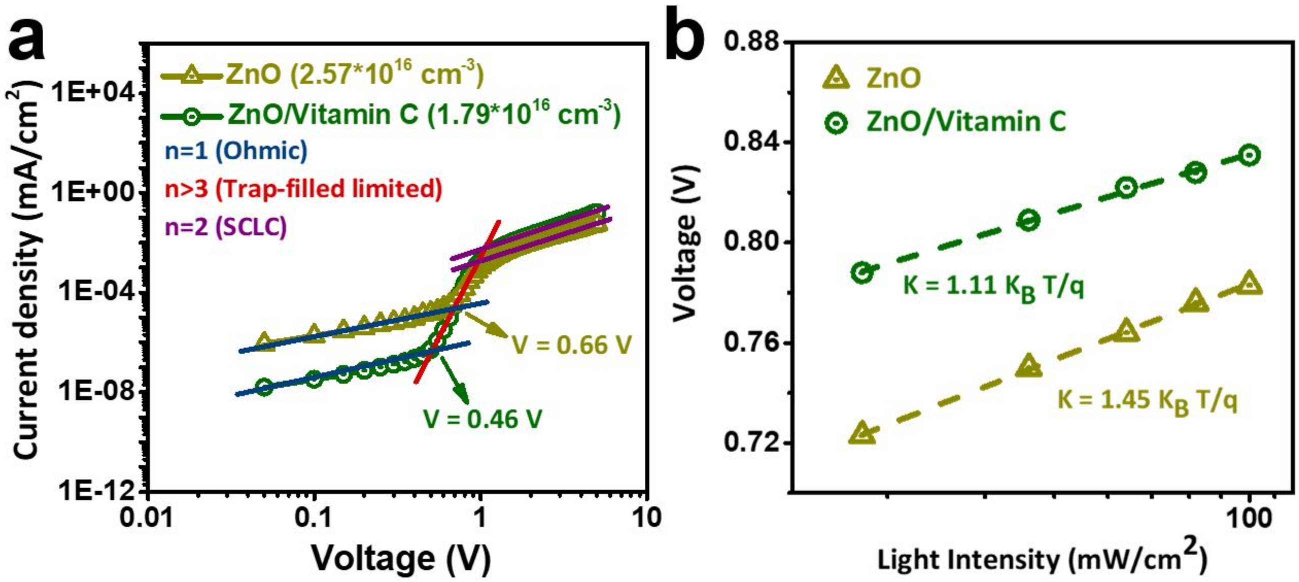

Figure S8. (a) Current-voltage traces and trap density of ZnO and ZnO/vitamin C. (b) V_{oc} as a function of light intensity of ZnO and ZnO/vitamin C devices. These devices were continuously illuminated with AM 1.5G simulated solar light at one-sun intensity for 120 hr in N_2 before measurement.

Table S3 Compared with the fresh and photoaging devices with trap density and the slope (K) of the relationship between V_{oc} and illumination intensity for the ZnO and ZnO/vitamin C devices.

	Fresh		Photo Aging	
Parameters	N_{trap}	K (K_B T/q)	N_{trap}	K (K_B T/q)
ZnO	2.38*10^{16} cm^{-3}	1.35	2.57*10^{16} cm^{-3}	1.45
ZnO/Vitamin C	1.75*10^{16} cm^{-3}	1.09	1.79*10^{16} cm^{-3}	1.11

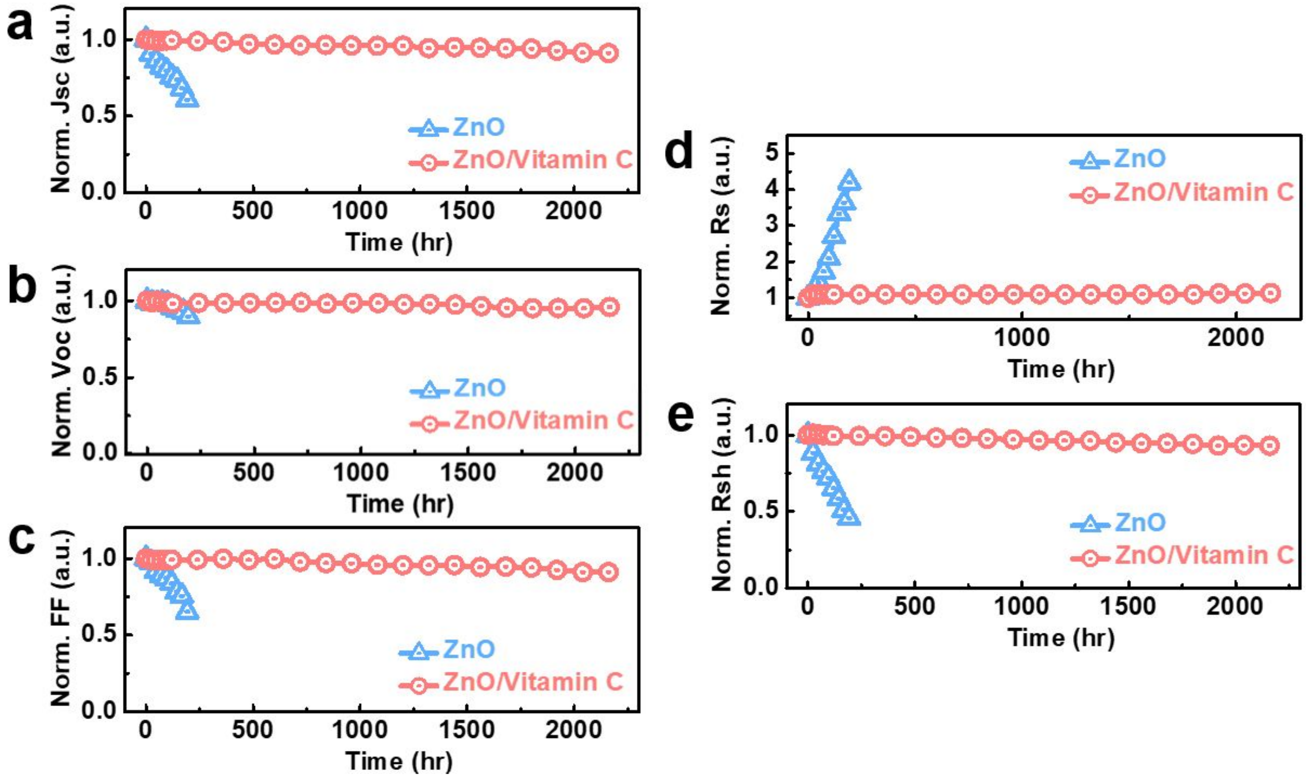

Figure S9. The evolutions of normalized (a) J_{sc}, (b) V_{oc}, (c) FF, (d) R_s, and (e) R_{sh} of the ternary OSCs under continuous one-sun illumination with an AM1.5G filter in N_2 condition.

Table S4 Photovoltaic performance of the inverted PSCs devices based on the NiO_x and NiO_x/vitamin C.

Parameters	V_{oc} (V)	J_{sc} (mA/cm^2)	Fill Factor (%)	PCE (%)	R_s (Ω·cm^2)	R_{sh} (kΩ·cm^2)
NiOx	0.97±0.02 (1.00)	22.20±1.85 (22.89)	74.79±1.40 (76.30)	16.30±1.22[a] **(17.47)**[b]	3.20±0.65 (2.85)	3.14±3.66 (3.52)
NiOx/Vitamin C (forward)	1.03±0.01 (1.03)	23.91±0.11 (23.90)	76.23±1.52 (77.31)	18.37±0.44[a] **(19.10)**[b]	2.82±0.11 (2.59)	3.47±2.89 (4.14)
NiOx/Vitamin C (reverse)	1.03	24.00	77.27	**19.00**[b]	2.81	4.43

a. The average PCE are based on 20 devices. b. Parentheses show the values from the champion devices.

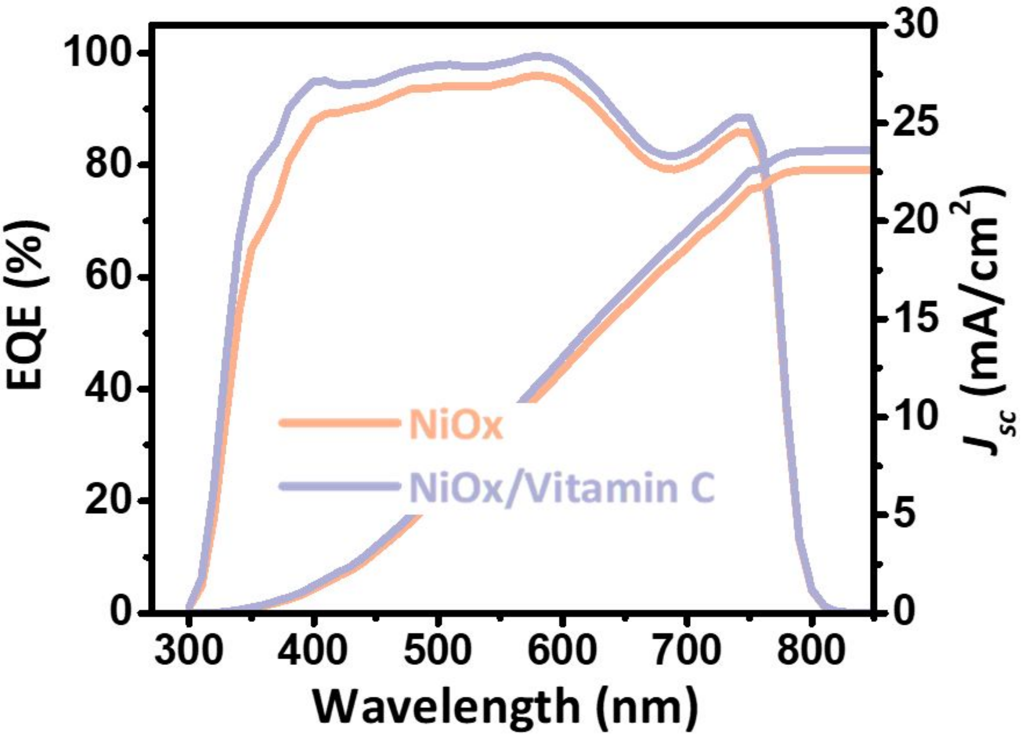

Figure S10. EQE spectra of our fabricated the inverted PSCs devices based on the NiO_x and NiO_x/vitamin C HTMs.

Table S5 The parameters τ_1 and τ_2 of biexponential decay fitting for the TRPL results.

Parameters	τ_1		τ_2	
	Lifetime (ns)	Content (%)	Lifetime (ns)	Content (%)
NiOx	4.58	71.37	10.74	28.62
NiOx/Vitamin C	2.44	60.42	6.23	39.57

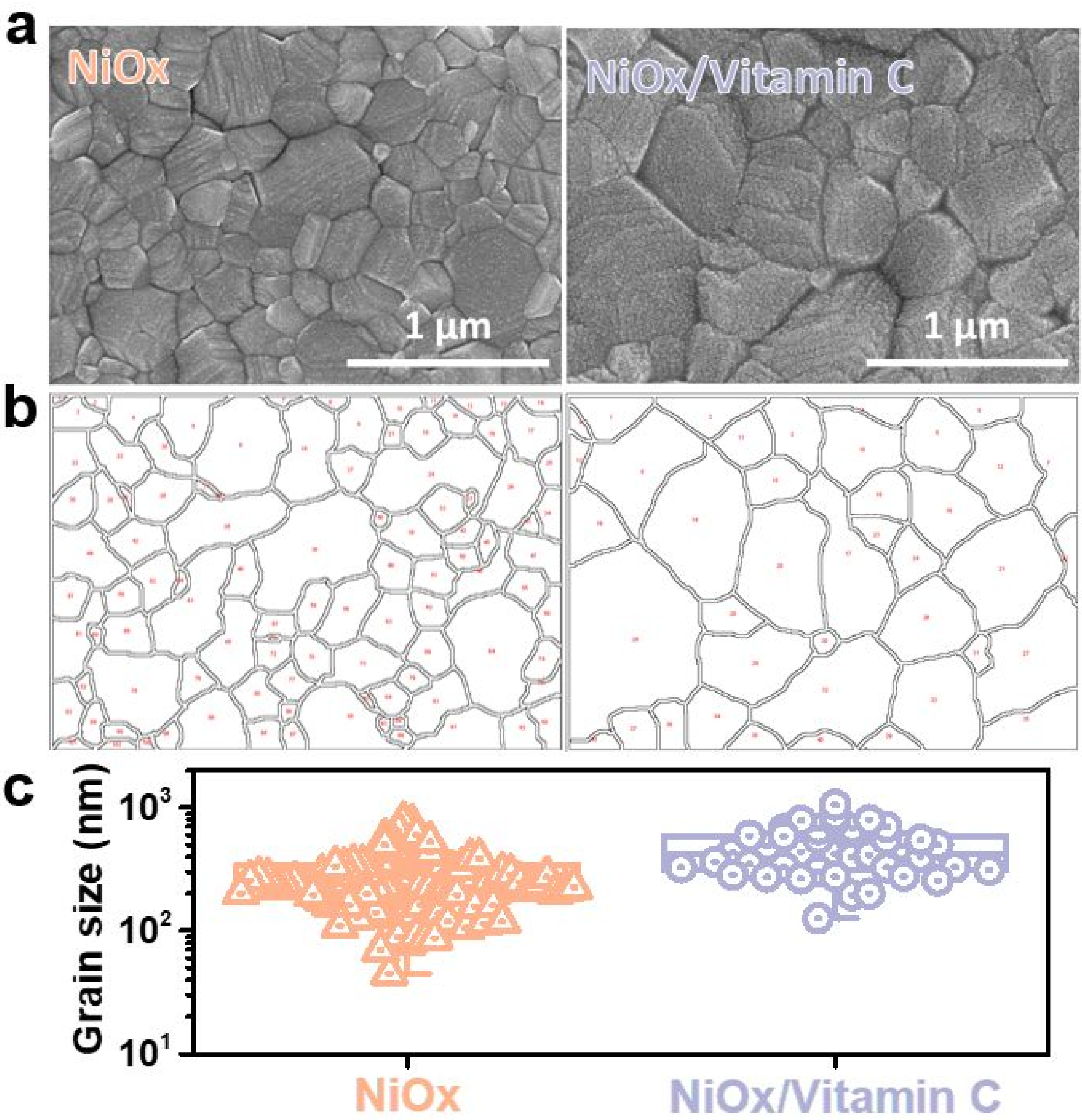

Figure S11. (a) The SEM top-view images of the perovskite film on NiO_x and NiO_x/vitamin C HTMs. (b) The graph is by Image J for determination of the of the perovskite film on NiO_x and NiO_x/vitamin C HTMs. (c) Perovskite's average grain size.

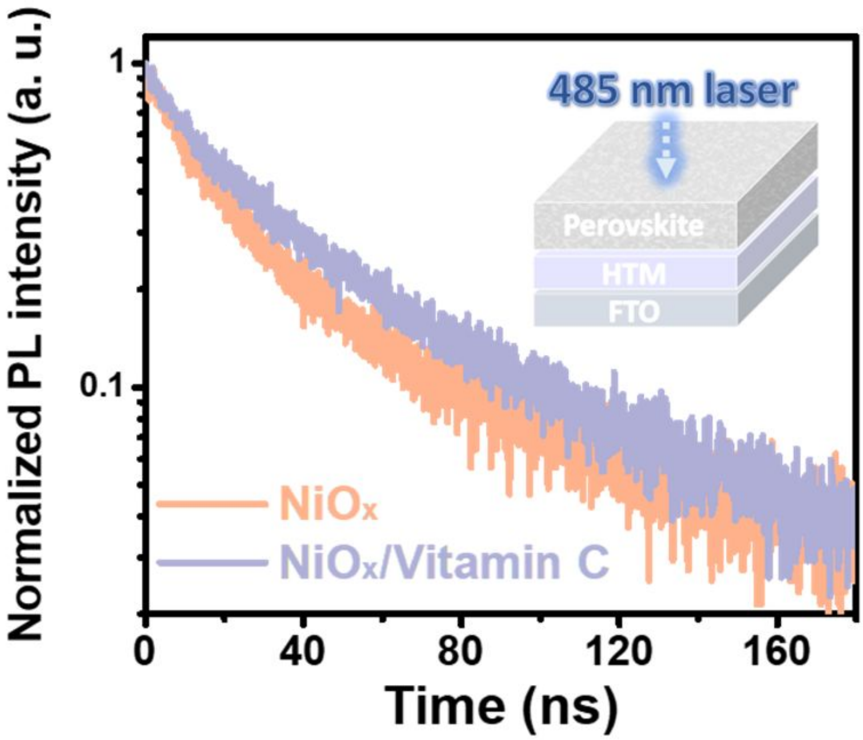

Figure S12. Time-resolved photoluminescence of perovskite on NiO_x and NiO_x/vitamin C HTMs.

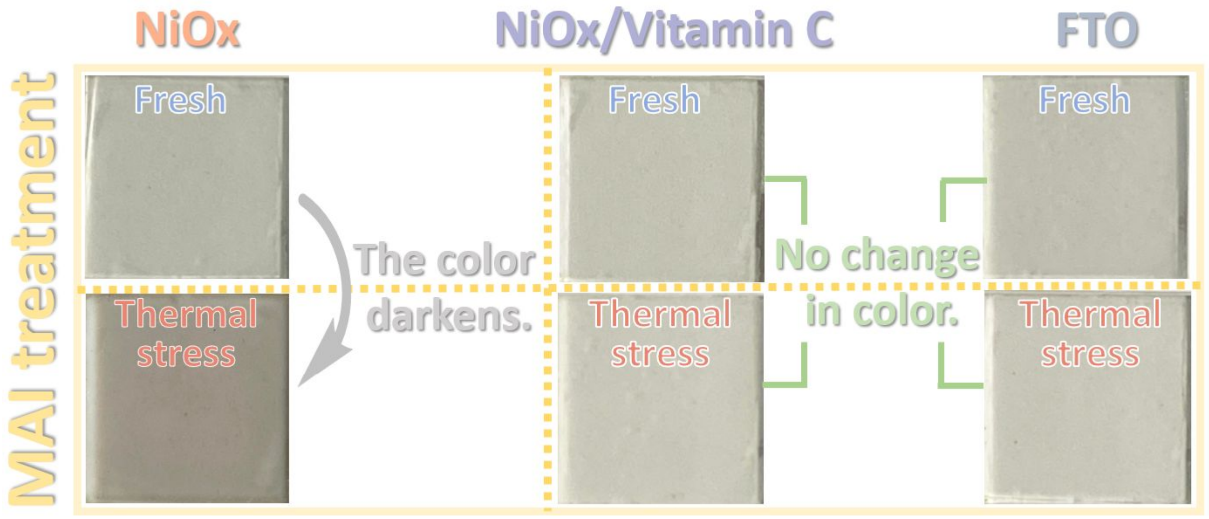

Figure S13. The color changes of MAI on ITO, NiO_x, and NiO_x/vitamin C substrates under thermal stress at 65 °C for 12 hours.

EXPERIMENTAL DETAILS

Materials. PM6 and Y6 were obtained from WAYS Technical Corp., Ltd. (Taiwan) and used as received without any additional purification. Chemicals were obtained from the following suppliers: L(+)-Ascorbic acid (vitamin C) was obtained from Acros Organics, MAI from Greatcell Solar Materials Pty Ltd., and lead(II) iodide (PbI_2) from Tokyo Chemical Industry Co., Ltd. Sigma-Aldrich provided nickel(II) chloride hexahydrate, lithium nitrate ($LiNO_3$), dimethyl sulfoxide (DMSO), molybdenum trioxide (MoO_3), [6,6]-Phenyl-C_{61}-butyric acid methyl ester ($PC_{61}BM$), polyethylenimine, methanol (MeOH), chloroform (CF), N,N-dimethylformamide (DMF), 1-chloronaphthalene (CN), diethyl ether, 2-methoxyethanol, chlorobenzene (CB), and ethanolamine.

Device fabrication. Inverted OSCs were fabricated with the device structure of ITO glass/ZnO or ZnO/vitamin C/active layer/MoO_3/Ag.[1] The ITO glasses underwent thorough cleaning through ultrasonic treatment with detergent, deionized water, acetone, and isopropanol, with each solvent being applied for 30 minutes. The substrates were then dried using N_2 flow and subjected to a plasma treatment for 10 minutes. A ZnO precursor solution was prepared by dissolving 100 mg of zinc acetate in 1 mL of 2-methoxyethanol, followed by the addition of 28 μL of ethanolamine. This precursor was spin-coated onto the ITO substrates at 4000 rpm for 30 seconds and then annealed at 220°C for 30 minutes in ambient air. A vitamin C solution with a concentration of 0.1 mg/mL was prepared in methanol (MeOH). The solution was spin-coated onto the ZnO ETL at 4000 rpm for 30 seconds and annealed at 110°C for 30 seconds inside a N_2 glovebox. The thickness of the vitamin C was estimated

using a Surface Profiler (α-stepper) to be around 1 to 2 nm. The precursor solution for the PM6:Y6 BHJ blend (1:1.2 weight ratio) was made in chloroform (CF) at a concentration of 16 mg/mL, with a 0.5% v/v CN additive (CF/CN = 99.5/0.5). The solution was stirred vigorously at 65°C for 12 hours in a N_2 glovebox. The PM6:Y6 BHJ solution was then spin-coated onto the ZnO or ZnO/vitamin C ETLs at 3000 rpm for 40 seconds, resulting in a film thickness of about 100 nm. The films were subsequently annealed at 110°C for 10 minutes in a N_2 glovebox. For the PM6:Y6:$PC_{71}BM$ BHJ blend (1:1.2:0.2 weight ratio), the solution was prepared in CF at a concentration of 18.7 mg/mL, with a CN additive (CF/CN = 99.25/0.75). The solution was stirred at 65°C for 12 hours in a N_2 glovebox. The PM6:Y6:$PC_{71}BM$ BHJ solution was spin-coated onto the ZnO or ZnO/vitamin C ETLs at 3000 rpm for 40 seconds, followed by annealing at 90°C for 10 minutes in a N_2 glovebox. The top electrode was formed by sequentially thermally depositing 8 nm MoO_3 and 100 nm Ag under high vacuum ($<10^{-6}$ Torr). The active area of the device was 0.07 cm^2.

To fabricate inverted PSCs, the device structure consisted of FTO glass/NiO_x or NiO_x/vitamin C/active layer/$PC_{61}BM$/polyethylenimine/Ag.[2] The FTO glasses were thoroughly cleaned by ultrasonic treatment in detergent, deionized water, acetone, and isopropanol, with each solvent applied for 30 minutes. After cleaning, the substrates were dried with N_2 flow and subjected to plasma treatment for 10 minutes. For the NiO_x precursor, 240 mg of nickel (II) chloride hexahydrate was dissolved in 10 mL of 2-methoxyethanol, with 63 μL of ethanolamine added, followed by the inclusion of lithium nitrate at a 5 molar% concentration. The resulting solution was filtered through a 0.22 μm

polytetrafluoroethylene (PTFE) filter and spin-coated onto the FTO substrates at 2000 rpm for 30 seconds. The FTO/NiO_x substrates were then dried in a N_2 glovebox at 150°C for 20 minutes and sintered in the atmosphere at 450°C using a tube furnace. The vitamin C solution was spin-coated onto the NiO_x ETL at 4000 rpm for 30 seconds and then annealed for 30 seconds at 100°C inside a N_2 glovebox. In a N_2 glovebox, the perovskite precursor solution was prepared by dissolving PbI_2 and MAI in a 1:1 molar ratio in a solvent mixture of DMF and DMSO (4:1, v/v) and stirring the solution at room temperature for 12 hours. The perovskite solution was then filtered through a 0.22 μm PTFE filter and spin-coated onto the NiO_x or NiO_x/vitamin C ETLs at 6000 rpm for 25 seconds. At the 6-second mark of spin-coating, 3 mL of diethyl ether was added dropwise as an anti-solvent. The films were annealed at 100°C for 1 hour and allowed to cool to room temperature. Afterward, a $PC_{61}BM$ solution (25 mg/mL in chlorobenzene) was spin-coated at 1000 rpm for 60 seconds in a N_2 glovebox to serve as the ETL. Polyethyleneimine was then spin-coated at 4000 rpm for 30 seconds. Finally, 100 nm of Ag was thermally evaporated under high vacuum ($<10^{-6}$ Torr) to form the top electrode. The active area of the device was 0.038 cm^2.

Characterization. The IR and UV-vis absorption spectra were acquired using a JASCO 4100 FTIR spectrometer and a JASCO V-670 spectrometer (JASCO, Tokyo, Japan). Photoluminescence spectra were recorded with an Edinburgh PLS 920 system employing time-correlated single photon counting (TCSPC) for PL decay analysis. A 485 nm laser (LDH-P-C-485, PicoQuant) excited the samples at

2.5 MHz, controlled by a PDL 800-B driver, with a 400 ns pulse duration at 25.4 μW cm^{-2}. The emitted PL signals were captured using a low-noise single-photon avalanche detector (ID-100-50, Becker & Hickl). The EQE was obtained using a QE-R monochromatic light source (Enlitech), calibrated against a silicon reference cell over the range of 300-800 nm. EIS was performed with a Metrohm Autolab PGSTAT 320N, applying a 15 mV AC perturbation from 1 MHz to 1 Hz under dark conditions at zero bias. XPS analysis was conducted with a Theta Probe AR-XPS using Al-Kα radiation ($h\upsilon$ = 1486.6 eV). XRD patterns were obtained with a Bruker D8 diffractometer using Cu-Kα radiation (λ = 1.5418 Å). The morphology of the perovskite film was analyzed with a Hitachi S-4800 SEM. *J-V* curves for OSCs were scanned with a Keithley 2400 source meter, scanning from -0.05 V to 1 V (0.01 V steps, 10 ms delay), whereas PSCs were measured from 1.1 V to -0.01 V under identical conditions. The photovoltaic performance was assessed under 100 mW cm^{-2} AM1.5G illumination using a Xenon-lamp solar simulator (EnliTech), calibrated with an SRC 2020 silicon solar cell. TGA was conducted on a HITACHI STA 7200, heating the samples from 25 to 800°C at a rate of 10°C/min under N_2.

REFERENCES

1. Chen, G.-L.; Wang, S.-H.; Tseng, K.-W.; Huang, C.-I.; Wang, L. Highly Stable Organic Solar Cells with Robust Interface Using Fullerenol as Molecular Linker. *J. Mater. Chem. A*, **2025**, *13*, 2668-2676.
2. Nie, W.; Tsai, H.; Blancon, J.-C.; Liu, F.; Stoumpos, C. C.; Traore, B.; Kepenekian, M.; Durand, O.; Katan, C.; Tretiak, S.; Crochet, J.; Ajayan, P. M.; Kanatzidis, M.; Even, J.; Mohite, A. D.Critical Role of Interface and Crystallinity on the Performance and Photostability of Perovskite Solar Cell on Nickel Oxide. *Adv. Mater.* **2018**, *30*, 1703879−1703887.
